# Characterization of Anti-Canine PD-1 Antibodies

**DOI:** 10.3390/cells15110966

**Published:** 2026-05-23

**Authors:** Colin J. Hartman, Petra Sergent, Anna Barbara Emilia Zimmermann, Olga R. Chávez-Alexander-Anderson, Luis A. Perez Alonso, Louise Lines, Juan Carlos Pinto-Cárdenas, Daniel Luna Dávalos, Anna M. Schmoker, Scott M. Palisoul, Johannes vom Berg, Xiaoxuan Ge, Jay L. Rothstein, Margaret E. Ackerman, Steven Fiering, Randolph J. Noelle, Hugo Arias-Pulido

**Affiliations:** 1 Department of Microbiology and Immunology, Geisel School of Medicine at Dartmouth, 621 Rubin Building—HB7936, 1 Medical Center Drive, Lebanon, NH 03756, USA; colin.j.hartman.gr@dartmouth.edu (C.J.H.); petra.sergent@dartmouth.edu (P.S.); janet.lines@dartmouth.edu (L.L.); margaret.e.ackerman@dartmouth.edu (M.E.A.); steven.n.fiering@dartmouth.edu (S.F.); randolph.j.noelle@dartmouth.edu (R.J.N.); 2 Clinic for Radiation Oncology and Medical Oncology, University Animal Hospital, Vetsuisse Faculty, University of Zurich, 8057 Zurich, Switzerland; annabarbaraemilia.zimmermann@uzh.ch; 3 Institute of Laboratory Animal Science, University of Zurich, 8952 Schlieren, Switzerland; johannes.vomberg@uzh.ch; 4 Villareal Garza, Hospital Veterinario, Monterrey 64390, Mexico; olgaalexaander@gmail.com (O.R.C.-A.-A.); luisalbertoperezalonso@gmail.com (L.A.P.A.); 5 DIAGSA, Diagnóstico de Salud Animal, Naucalpan 53910, Mexico; juancarlospintoc@gmail.com; 6 VETCONNECT Diagnóstico por Imagen, Monterrey 64780, Mexico; drvet.daniel.luna@gmail.com; 7 Dartmouth Cancer Center, Geisel School of Medicine at Dartmouth, Lebanon, NH 03756, USA; anna.m.schmoker@dartmouth.edu; 8 Center for Clinical Genomics and Advanced Technology, Department of Pathology and Laboratory Medicine at Dartmouth Hitchcock Health, Lebanon, NH 03756, USA; scott.m.palisoul@hitchcock.org; 9 Thayer School of Engineering, Dartmouth College, Hanover, NH 03766, USA; grace.ge@dartmouth.edu; 10 Lifordi Immunotherapeutics, Lebanon, NH 03756, USA; jay.l.rothstein@dartmouth.edu; 11 Canine Cancer Alliance, Bellevue, WA 98005, USA

**Keywords:** antibody, canine, cancer, mammary tumors, mast cell tumors, therapeutic antibodies, preclinical trial

## Abstract

Cancer is a leading cause of death in dogs, and incidence rates in dogs exceed those in humans. Current therapeutic options for canine cancer patients remain limited, with most treatments focused on palliative care. Immune checkpoint inhibitors such as anti-PD-1, anti-PD-L1, and anti-CTLA-4 antibodies that have transformed cancer therapy and expanded the therapeutic options in humans could offer the same clinical benefit in canine cancer patients. This study details the engineering and functional characterization of mouse and chimeric mouse–canine anti-canine PD-1 (cPD-1) monoclonal antibodies. We demonstrate that anti-cPD-1 antibodies block the interaction between cPD-1 and its ligand cPD-L1, thereby inhibiting this immune signaling pathway. In a proof-of-concept study in seven companion canine cancer patients, intratumoral therapy with the lead anti-cPD-1 antibody (HugPetmab) was safe, well-tolerated, had no observed adverse events, and showed evidence of tumor control in a subset of injected tumors. These findings support the potential of HugPetmab antibody as an immunotherapeutic option for treating canine cancer patients.

## 1. Introduction

It is estimated that ~1.7 million humans (~500 per 100,000 persons) and ~4.2 million dogs (~5300 per 100,000 dogs) were diagnosed with cancer in the USA in 2011 [1]. The current incidence in dogs is believed to be higher [1,2]. Although current statistics are not available for dogs, there were ~89 million dogs in the USA in 2020; one in four dogs will, at some stage in their life, develop neoplasia, and almost half of dogs over the age of 10 will develop cancer [2]. Crude estimates of cancer incidence indicate that ~6 million new cancer diagnoses are made in dogs each year in the USA [3]. Retrospective studies suggest that cancer is the most common cause of death in dogs with an estimated rate of 30% [4]. For comparison, cancer is the second leading cause of death in humans in the USA, second to vascular disease, with an estimated rate of ~18% [5,6]. When adjusted for expected lifespan, cancer deaths occur roughly at the same stage of life span in both species [7].

Despite a 10-fold higher cancer incidence rate per year of life in dogs than in humans, dogs do not have many therapeutic options, and most dogs suffer disease progression with palliative care being the only treatment option available. Immunotherapy has changed the therapeutic treatment in several human tumors. In particular, antibody-based targeting of programmed cell death 1 (PD-1) and the cytotoxic T-lymphocyte antigen 4 (CTLA-4) immune checkpoint inhibitors (ICIs) have reshaped immunologic approaches to the treatment of various human tumor types [8,9,10,11].

PD-1 is a transmembrane protein receptor that functions as a major negative immune regulator, controlling T cell activation, T cell exhaustion, T cell tolerance, and resolution of inflammation [12,13]. It is expressed primarily on the surface of activated T cells, Tregs, exhausted T cells, B cells, activated monocytes, dendritic cells, natural killer (NK) cells and natural killer T (NKT) cells [14,15]. The interaction of PD-1 with its primary ligand PD-L1 inhibits CD4^+^ and CD8^+^ T-cell effector functions, including cytotoxic activity, cell proliferation, migration, and cytokine secretion, and therefore protects the host from unrestrained immune responses [12,13,16]. In the light of good responses to ICIs in some human tumors, equivalent monoclonal antibodies (mAbs) to treat canine cancer patients have been sought [17,18], including development of anti-canine PD-1/PD-L1 [19,20,21,22,23,24,25,26,27,28], and anti-canine CTLA-4 [29,30,31] antibodies.

We previously demonstrated that intratumoral (IT) therapy with a mouse anti-canine PD-1 antibody was safe, effective, and did not cause immune-related adverse events (irAEs) in companion dogs diagnosed with spontaneous canine mammary cancer (CMC) [32]. In the present study, we report characterization of the mouse anti-canine PD-1 antibody and optimization of the therapeutic potential of the parental mouse antibody by caninization of four mouse-derived anti-cPD-1 mAbs and evaluation of the lead antibody (HugPetmab) in a proof-of-concept trial in seven canine cancer patients. Our study suggests that IT HugPetmab monotherapy is safe, well-tolerated, does not cause irAEs, and it has a positive effect on controlling tumor burden in injected tumors. This work supports emerging efforts to expand treatment options for canine oncology patients and open new avenues to model current PD-1-based immunotherapy in human cancers to identify effective immunotherapeutic combinations in a clinically relevant immunocompetent animal.

## 2. Materials and Methods

### 2.1. Cell Lines

Chinese hamster ovary (CHO) cells and human embryonic kidney (HEK293) cells (ATCC, Manassa, VA, USA) were maintained in D10 complete medium (DMEM supplemented with 10% fetal bovine serum, 100 units/mL penicillin, 100 μg/mL streptomycin, and 55 μM 2-mercaptoethanol) and cultured at 37 °C in a humidified incubator with 5% CO_2_. ExpiCHO-S™ cells (Thermo Fisher Scientific, Waltham, MA, USA) were cultured in ExpiCHO-S^TM^ expression media (Thermo Fisher Scientific) in an incubator with 8% CO_2_, at 37 °C, and shaking. To monitor cell density and viability, an automated cell counter TC20 (Bio-Rad, Hercules, CA, USA) was used routinely, and all cells were maintained over 99% viability during culturing. The ExpiCHO cells were negative for mycoplasma using the mycoStrip test (InvivoGen, San Diego, CA, USA).

### 2.2. Identification of Mouse Anti-Canine PD-1 mAb

All hybridoma generation and handling was performed by a contract research organization (GenScript; Piscataway, NJ, USA). Briefly, mAbs were generated via conventional hybridoma procedures using BALB/c mice immunized with the extracellular domain (ECD) of canine PD-1 (cPD-1; Cat. 70109-D08H; Sino Biological, Wayne, PA, USA; Supplementary Figure S1). Splenocytes were isolated from the immunized mice and then fused with SP2/0 myeloma cells. Cell fusions were performed by electro fusion and plated onto 96-well plates. Supernatants were screened by ELISA with PD-1 ECD protein and by flow cytometry using HEK293 cells overexpressing canine PD-1. From among five isolated clones, clone 77A6H9 (H9) was selected as the lead antibody for further studies. Total RNA was extracted from H9 producing hybridoma cells using TRIzol Reagent (Thermo Fisher Scientific) and reverse-transcribed into cDNA with either isotype-specific antisense or universal primers following the PrimeScript 1st Strand cDNA Synthesis Kit (Takara San Jose, CA, USA) protocol. Antibody heavy- and light-chain fragments were amplified using GenScript’s rapid amplification of cDNA ends (RACE) protocol, cloned into standard cloning vectors, and screened by colony PCR. Consensus sequences were obtained, and the variable regions of clone H9 were synthesized as gene fragments for subcloning into an expression plasmid. H9 antibody was characterized by Sodium Dodecyl Sulfate-Polyacrylamide Gel Electrophoresis (SDS-PAGE); dynamic light scattering (DLS) was used to assess the purity and aggregation state of antibodies; size-exclusion chromatography (SEC) and high-performance liquid chromatography (HPLC) were performed to assess the purity and size distribution of the antibody; and antibody sequences were confirmed by liquid chromatography tandem mass spectrometry (LC-MS).

### 2.3. Characterization of H9 mAb

SEC and SEC-HPLC were used to isolate H9 and remove aggregates. The sample was applied to a Cytiva Superdex 200 pg column (Cytiva, Wilmington, DE, USA) on an AKTApure system and eluted in a phosphate buffer (22 mM Na_2_PO_4_, 9.9 mM NaH_2_PO_4_, 136 mM NaCl, pH 7.0). The eluted fraction was analyzed on an Agilent AdvanceBio SEC 300A HPLC column (Agilent, Santa Clara, CA, USA) using 50 mM phosphate buffer, pH 6, 200 mM Arginine, and 5% isopropyl alcohol as the running buffer to estimate final purity. A second HPLC analysis was conducted using phosphate buffer as the running buffer to compare peak profiles. The H9 mAb purity was 95% and the endotoxin levels were <0.5 EU/mg. The SEC analysis was performed by a contract research organization (Sino Biological, Houston, TX, USA).

For the LC-MS characterization, H9 antibody was either digested in solution or via single-pot solid-phase-enhanced sample preparation (SP3) [33]. For in-solution digestion, antibodies were denatured in 8 M urea and reduced/alkylated in 2 mM dithiothreitol and 4 mM iodoacetamide prior to 5× dilution in 50 mM HEPES, pH 8.5 and the addition of trypsin at a 1:40 ratio of enzyme to antibody. For SP3 digestions, Sera-Mag SpeedBeads (E3 and E7) (Cytiva,) were added to antibodies at a 1:10 protein:bead ratio prior to dilution to 50% ethanol. Mixtures were shaken at 1000 rpm for 5 min to bind proteins to beads. The beads were then washed four times with 80% ethanol before resuspension in 20 mM ammonium bicarbonate with endoproteinase Glu-C at a 1:20 ratio of enzyme to antibody. Digestions were incubated at 30 °C overnight, then acidified with trifluoroacetic acid, brought to 8% methanol, and desalted over micro hydrophilic–lipophilic balanced elution plates (Waters Corporation, Milford, MA, USA).

Peptides were separated across a 45 min gradient of 4–32% acetonitrile in 0.125% formic acid over a 32 cm × 100 µm column (ReproSil-Pur 120 C18-AQ 3 µm; Dr. Maisch, Ammerbuch, Germany) with an Easy-nLC 1000 (Thermo Scientific) and electrosprayed (1.91 kV, 250 °C) into a Q Exactive Plus (Thermo Fisher Scientific) mass spectrometer with a Nanospray Flex source (Thermo Fisher Scientific). Precursor ion scans (350–1500 *m*/*z*) were obtained at 70,000 resolution in centroid. Fragment ion scans were acquired in data dependent mode (26% NCE, 1.0 *m*/*z* isolation, 17,500 resolution, 15 s dynamic exclusion).

Raw data were searched against a target-decoy database containing the antibody sequences and human proteome (Uniprot) or the antibody sequences alone using Comet [34], permitting a mass tolerance of ±5 ppm, three missed cleavages, and the following modifications: methionine oxidation, cysteine carbamidomethylation, lysine methylation, and N-terminal glutamine-to-pyroglutamic acid. Spectra of post-translational modification sites and non-tryptic/GluC cleavages were manually validated.

For identification of antibody species, Coomassie-stained bands of interest were excised and diced into 1 mm cubes and submerged in DI water. Gel pieces were destained in 20 mM ammonium bicarbonate (AmBic) and 50% acetonitrile for 30 min at 37 °C before complete dehydration in 100% acetonitrile. Disulfide bonds were reduced with 20 mM dithiothreitol in 20 mM AmBic for 30 min at 56 °C. Gel pieces were dehydrated with acetonitrile prior to incubation in 55 mM iodoacetamide in 20 mM AmBic for 45 min at room temperature in the dark. Gel pieces were washed in 20 mM AmBic and fully dehydrated in acetonitrile twice. Gel pieces were rehydrated in 7.5 ng/μL sequencing-grade, modified trypsin (V5111, Promega, Madison, WI, USA) in 20 mM AmBic and incubated overnight at 37 °C. Peptides were extracted in a solution of 2.5% formic acid in 50% acetonitrile by centrifugation at 18,000× *g* prior to complete dehydration in acetonitrile. Supernatants post-digestion were combined and dried in a vacuum centrifuge. Peptides were resuspended in 0.1% formic acid and desalted over C18 ZipTips (Millipore, Rockville, MD, USA).

Peptides were separated across a 45 min gradient of 0–37% acetonitrile in 0.125% formic acid over a 25 cm × 100 µm column (ReproSil-Pur 120 C18-AQ 1.9 µm, Dr. Maisch) with a Vanquish Neo liquid chromatography system (Thermo Scientific) and electrosprayed (2.0 kV, 250 °C) into an Orbitrap Fusion Lumos (Thermo Scientific) mass spectrometer with a Nanospray Flex source (Thermo Scientific). Precursor ion scans (350–1500 *m*/*z*) were obtained at 120,000 resolution in centroid. Fragment ion scans were acquired in a data-dependent mode (28% NCE, 1.0 *m*/*z* isolation, 15,000 resolution, 18 s dynamic exclusion).

Raw data were searched against a target-decoy database containing the engineered protein sequence and the *C. griseus* proteome (Uniprot) using Comet [34], permitting a mass tolerance of ±5 ppm, three missed cleavages, and the following variable modifications: methionine oxidation, cysteine carbamidomethylation, and cysteine acrylamidation. Relative abundance of protein species were normalized using the IBAQ method [35] to approximate relative abundances.

### 2.4. Evaluation of Functional Activity of H9 in Canine Peripheral Blood Mononuclear Cells (cPBMCs)

Whole blood was collected from healthy canine donors in EDTA anticoagulant-containing tubes. Healthy cPBMCs were isolated from the obtained blood samples in accordance with Zimmermann et al. (2025) [36]. The samples were initially preserved in a freezing container at −80 °C for 2 days and then transferred to −150 °C for long-term storage until further analysis. For analysis or stimulation of cryopreserved PBMCs, thawed aliquots were slowly diluted in warm complete medium containing RPMI1640 (Sigma-Aldrich, St Louis, MO, USA), 10% FBS (Sigma-Aldrich), GlutaMAX (2 mM), penicillin-streptomycin (100 units; Thermo Fisher), sodium pyruvate (1 mM), non-essential amino acids (0.1 mM) and HEPES buffer (25 mM; all from Thermo Fisher). PBMCs were washed with warm medium and used in the assays at described concentrations.

For the assessment of functional activity of H9 antibody, 2 × 10^5^ cells were distributed per well. Cell stimulation and activation were performed as previously described [36]. Briefly, cells were stimulated with 50 ng/mL Staphylococcal Enterotoxin B (SEB; Toxin Technology, Sarasota, FL, USA) and 1–30 µg/mL anti-canine PD-1 antibody. Each stimulation was carried out in biological duplicates on sterile 96-well plates. The samples were incubated at 37 °C for 72 h. After incubation, the plate was centrifuged, and the supernatant was collected and stored at −20 °C.

For the evaluation of canine interferon gamma production (cIFN-γ) in cPMBCs, all ELISA assays were performed on high binding plates (Sigma-Aldrich). Each supernatant sample was analyzed as a technical duplicate. Canine IFN-γ was quantified using a Canine IFN-γ ELISA development kit (Mabtech, Nacka Strand, Sweden). The absorbance levels were measured with a SPARK plate reader (TECAN, Männedorf, Switzerland).

### 2.5. Detection of Mouse Anti-Canine PD-1 Binding by Immunohistochemistry (IHC)

HEK293 cells were transfected using a pcDNA3.1 expression plasmid encoding the full-length cPD-1 sequence and lipofectamine 3000 transfection reagent (Thermo Fisher Scientific), and Opti-MEM (Thermo Fisher Scientific) to generate HEK293 cells expressing surface cPD-1. HEK 293-cPD-1+ cells (~5000 cells) were resuspended in phosphate-buffered saline (PBS) containing 5% of fetal bovine serum and deposited on positive glass slides (resembling fresh-frozen samples) or fixed with 10% neutral buffered formalin, prepared in Histogel (Thermo Fisher Scientific), and embedded in paraffin to resemble paraffin-embedded tissues. In addition, normal canine tonsils embedded in paraffin were also used to detect cPD-1 by IHC. Briefly, cells in glass slides or tissue samples cut at 4 microns were air dried at room temperature. Slides were baked at 60 °C for 30 min prior to being loaded onto the Leica Bond Rx autostainer (Danvers, MA, USA). Automated protocol includes paraffin dewax with Bond Dewax Solution, antigen retrieval using Bond Epitope Retrieval Solution 2 (pH 9) incubated for 40 min at 100 °C. Tissue was blocked with 2.5% of horse/bovine serum albumin solution for 3 min at ambient temperature prior to primary antibody incubation. Slides were removed from the autostainer to allow for overnight incubation with H9 antibody (~50 µg/mL of each individual antibody) at 4 °C in a humidity tray. The next day, slides were rinsed in 10× Bond Wash Buffer and loaded onto the Bond Rx for visual detection. The automated protocol for day two includes incubating slides with mouse-on-canine HRP Polymer (MC541; Biocare, Pacheco, CA, USA) for 55 min at ambient temperature before visual detection is completed using a Leica Bond Refine Detection System (Leica DS9800) with 3,3′-diaminobenzidine chromogen and hematoxylin counterstain.

### 2.6. Sequence Analysis of PD-1 Across Species and Caninization of the Mouse mAb

Sequence alignments were performed for the PD-1 protein from murine (UniProt: Q02242), canine (UniProt: A0A8I3PR61), feline (UniProt: M3WAP8), and human (UniProt: Q15116) species. To assess sequence conservation and identify differences in functional regions across species, the sequences were aligned using Clustal Omega (v. 1.2.4) within the Jalview software environment (v. 2.11.1.0).

Sequences for canine IgG subclasses were obtained from the IMGT database (*Canis lupus familiaris*, IGHG1 and IGHG4). Based on the parental mouse IgG1 (clone 77A6H9; or H9), we engineered four novel mouse–canine chimeric mAbs, each with the same variable domains but differing constant domains. Expression plasmids were assembled through High Fidelity DNA cloning following the manufacturer’s protocol (NEBuilder^®^ HiFi DNA Assembly E2621X), which fused the H9 variable domain and, in some cases, the CH1 region of the mouse antibody (H9) to either the hinge region of a canine IgG1 antibody or the CH1 region of canine IgG1 or IgG4. Additionally, the CH2 and CH3 regions of a canine IgG4 antibody were incorporated to reduce Fc effector functional activity. Sequencing was performed to confirm the DNA sequence of the plasmids.

Plasmid DNA encoding antibody heavy chain (HC) or light chain (LC) was transformed in *E. coli* DH5α. The plasmids were purified with the Pure Link™ Expi Endotoxin-Free Maxi Plasmid Purification kit (Thermo Fisher Scientific, A31217). DNA concentration and quality was determined using a DeNovix DS-11FX+ Microvolume Spectrophotometer (DeNovix, Wilmington, DE, USA). DNA sequences were verified through Sanger sequencing conducted by the Dartmouth College Sequencing Core.

### 2.7. Production of Chimeric cPD-1 Antibodies

From the parent mouse antibody H9, four chimeric mouse–canine antibodies (referred to as 77A6H9c.1, 77A6H9c.2, 77A6H9c.3, and 77A6H9c.4) were produced using ExpiCHO suspension cultures, with transient transfection of the heavy and light chain plasmids following the manufacturer’s protocol (Thermo Fisher Scientific). Briefly, cells were routinely cultured in ExpiCHO™ Expression Medium at 37 °C, 8% CO_2_, and 125 rpm. For specific ExpiCHO transfection protocols, the temperature was shifted to 32 °C while maintaining 8% CO_2_. Transfections were performed using the ExpiFectamine™ CHO Transfection Kit with enhancers added 18–20 h post-transfection. ExpiCHO-S™ cells were subcultured to a cell density of 6–10 × 10^6^ cells/mL with >98% viability and adjusted to 6 × 10^6^ cells/mL immediately before transfection. A total DNA concentration of 1.0 µg/mL of cell culture was used, with a 1:1 or 3:2 plasmid ratio of light chain to heavy chain. DNA and ExpiFectamine™ CHO Reagent were diluted in Opti-MEM™ medium and mixed thoroughly before adding to the culture. On the first day post-transfection, ExpiFectamine™ CHO enhancer and ExpiCHO feed were added per the manufacturer’s instructions, with a second feed added on day five for high-titer batches. Supernatant was harvested 14 days post-transfection and antibodies were purified by gravity or fast protein liquid chromatography columns with protein G sepharose resin (Cytiva, Uppsala, Sweden). Purified antibodies were dialyzed and concentrated using Amicon Ultra-15 regenerated cellulose filters (Merck Millipore, Tullagreen, Ireland) and filtered through 0.2 µm filters (Thermo Fisher Scientific). Endotoxin levels were monitored and, if necessary, reduced using Q-sepharose resin (Cytiva), ensuring endotoxin levels remained <0.30 EU/mg. Based on expression and activity profiles, 77A6H9c.1 (HugPetmab) was chosen to be used in further studies. The antibody sequence was confirmed by LC-MS.

### 2.8. PD-1 Binding Assay

Biotinylated cPD-1 (Sino Biological, catalogue # 70109-D27H-B) was conjugated to M270 Streptavidin Dynabeads (Thermo Fisher Scientific) following the manufacturer’s protocol. The beads were incubated with varying concentrations of each anti-cPD-1 antibody at 4 °C for 1 h, and binding was detected using either a goat anti-mouse kappa-FITC (SouthernBiotech (Birmingham, AL) for antibodies c.1 (HugPetmab), c.3, and H9, or a goat anti-canine IgG(H+L)-FITC (SouthernBiotech) for antibodies c.2 and c.4 via flow cytometry on an Agilent NovoCyte Advanteon machine (Agilent Technologies, Santa Clara, CA, USA).

### 2.9. PD-1/PD-L1 Competition Assay

Biotinylated cPD-1 (Sino Biological, catalogue # 70109-D27H-B) was conjugated to M270 Streptavidin Dynabeads (Thermo Fisher Scientific) following the manufacturer’s protocol. Since both cPD-1 and cPD-L1 (Sino Biological, catalogue # 70110-D08H) had His tags, which was the method of detection, the cPD-1 beads were blocked for two hours at 4 °C with a non-fluorescent anti-His antibody (GeneTex, Irvine, CA, USA). Following blocking and washing, the conjugated beads were incubated with cPD-L1 at a concentration of 15 µg/mL and varying concentrations of HugPetmab and H9 antibodies for 1 h at 4 °C. Anti-PD-1 and PD-L1 antibodies were added at the same time. PD-1/PDL1 binding was detected using an anti-His antibody (Thermo Fisher Scientific). The flow cytometry readout was measured on an Agilent NovoCyte Advanteon machine.

### 2.10. Canine Safety Studies

A phase I non-randomized, unblinded, single-center trial to evaluate the safety and tolerability of HugPetmab alone in a neoadjuvant setting was performed in client-owned companion canine cancer patients. The standard of care for both CMC and MCT patients is surgery and, as needed, adjuvant chemotherapy. In this neoadjuvant study, we took advantage of ‘the window of opportunity’ of four weeks to evaluate our HugPetmab antibody followed by the standard of care, which was surgery and, as recommended by the attending veterinarian, adjuvant therapy. Seven companion pets with canine mammary tumors (CMTs; *n* = 3) or canine mast cell tumors (MCTs; *n* = 4) were enrolled in a proof-of-concept, open-label study performed at Villareal Garza Hospital Veterinario, Monterrey, Nuevo Leon, Mexico, and Diagnóstico de Salud Animal, Naucalpan, Mexico. All patients’ owners provided written informed consent. The study was approved by the Internal Committee for the Care and Use of Animals, Faculty of Veterinary Medicine and Zootechnics of the National Autonomous University of Mexico (Protocols #153 and #199). Client-owned dogs with histologically and cytology-based confirmed diagnosis of CMT and MCT with a tumor mass of at least 1.5 cm in any length, with or without metastatic disease, were eligible for enrollment. All patients were required to have a good performance status (modified Eastern Cooperative Oncology Group criteria 0–1) and had not received prior systemic therapy within 4 weeks of trial initiation. Inclusion criteria are described in the Supplementary File. The characteristics of seven individual dogs are described in Table 1. The clinical staging system, histopathological classification of tumors, and the histological grade of malignancy were evaluated as described elsewhere [37,38,39,40,41,42].

The primary objective of this study was to determine the safety profile and tolerability of neoadjuvant IT HugPetmab therapy. Secondary endpoints included irAEs and quality of life (QOL). The largest tumor mass in each patient was selected as the target tumor (injected) for IT injections. Other mammary nodules present in the same and contralateral chains in CMT or in any part of the MCT patients were either observed to evaluate systemic impact on noninjected nodules in the same canine patient or treated in a similar manner as the largest mass. Based on a previous study with the mouse H9 antibody [32], we used 0.5 mg for tumors of ≤4 cm in diameter and 1.0 mg for tumors > 4 cm. The final antibody dilution in PBS ranged from 0.3 to 1.0 mL and was administered either as a radial injection or injected in various sides of the tumor depending on the tumor size with the goal of irrigating the tumor area as much as possible. Companion dogs were treated with IT HugPetmab once a week for four weeks. After the four weeks of treatment, patients underwent planned surgery (described in the Supplementary File) with adjuvant therapy as recommended by the attending veterinary doctor.

### 2.11. Safety Evaluation

To evaluate systemic changes and track irAEs, hemograms were performed weekly. After IT injections, each canine patient was closely observed by the attending veterinarian for ~4 h in the veterinary clinic with follow-up three days later in the clinic. In addition, dogs were observed daily by owners to detect possible irAEs using a preestablished QOL survey [43], which was reviewed by the attending veterinarian prior to planned weekly treatment. The evaluation of hematological and other adverse events related to immunotherapy was conducted per the Veterinary Cooperative Oncology Group criteria (Version 2) [44].

A blood sample was collected from each dog prior to any injections to evaluate changes induced by HugPetmab immunotherapy. Hematological analyses were conducted using a standard hematology analyzer IDEXX^®^ Procyte Dx (IDEXX Laboratories, Westbrook, ME, USA). The blood panel included analysis of erythrocytes, hemoglobin, hematocrit, total leukocytes, neutrophils, monocytes, lymphocytes, eosinophils, reticulocytes and platelets.

### 2.12. Tumor Response Evaluation

The tumor response to the IT treatment was evaluated once or twice a week during the treatment period by measuring the tumor volume (Tv) using the formula Tv = 0.5 × long axis × (short axis)^2^. The percentage of change in tumor growth (%TG) was estimated as %TG = 100 × (final Tv − initial Tv)/initial Tv). All volumetric measurements were reported in cubic centimeters (cm^3^). Taking D0 as the reference, responses were defined as complete response (CR) when there was disappearance of all target lesions; partial response (PR), when at least a 30% decrease of target lesions occurred; progressive disease (PD), when at least a 20% increase in the target lesions occurred or one or more new lesions appeared; and stable disease (SD), when neither sufficient shrinkage to qualify for PR nor sufficient increase to qualify for PD was observed.

### 2.13. Statistics

Tumor size changes over time were evaluated using the Friedman test for repeated measures. Analyses were performed considering all tumors together and stratified by tumor type (CMT and MCT). Additionally, individual linear regression analyses were performed for each tumor to assess temporal trends in tumor size across follow-up time points, using the start of treatment (D0) as the baseline. Half maximal effective concentration (EC50) values were determined by fitting dose–response data to a sigmoidal four-parameter logistic model using nonlinear regression in GraphPad Prism v11.0.2 (GraphPad Software, San Diego, CA, USA). *p* values < 0.05 were considered significant.

## 3. Results

### 3.1. Identification and Characterization of a Lead-Candidate Mouse Anti-Canine PD-1 mAb

Applying the hybridoma antibody generation and screening approach (Figure S1), five individual mouse cPD-1 mAbs were isotyped and prepared as purified IgG for binding and functional studies. While some similarities were found between clones in binding and IFN-γ assays, clone 77A6H9 (H9) was selected as the lead antibody due to having the most consistent dose-dependent IFN-γ increase and highest binding affinity and will be described here.

An SDS-PAGE analysis shows the presence of a band at protein of approximately 150 kD, consistent with the predicted molecular weight of 146.8 kDa (Figure S2A). The DLS data revealed a single clean peak with a hydrodynamic radius of 5.4 nm, consistent with the estimated size by radius of the antibody monomer (Figure S2B). The SEC-HPLC chromatogram revealed a single sharp dominant peak with two minor second peaks indicating that H9 exists predominantly as a monomeric species, with no detectable aggregates or fragments (Figure S2C). The antibody sequence was confirmed by liquid chromatography-tandem mass spectrometry (LC-MS). Together, these data suggest that H9 is structurally intact, monodisperse, and exists primarily as a monomeric species.

**Figure 1:**
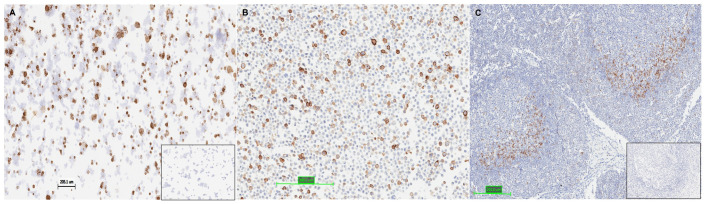
H9 binds to native canine PD-1 molecule. The transiently transfected HEK293 cells show the presence of canine PD-1 detected with H9 antibody by standard IHC assay in a cytospin format (resembling fresh-frozen tissues); (**A**) or embedded in paraffin (resembling formalin-fixed paraffin tissues); (**B**), and normal canine tonsils embedded in paraffin (**C**). Insets show a negative control using a murine IgG isotype control antibody.

Next, IHC assays indicated that the H9 antibody bound to PD-1 expressed on the surface of transiently transfected HEK293 cells as well as in canine tonsils (Figure 1A–C), indicating that H9 antibody binds to native PD-1 expressed by immune cells in canine tissues.

**Figure 2:**
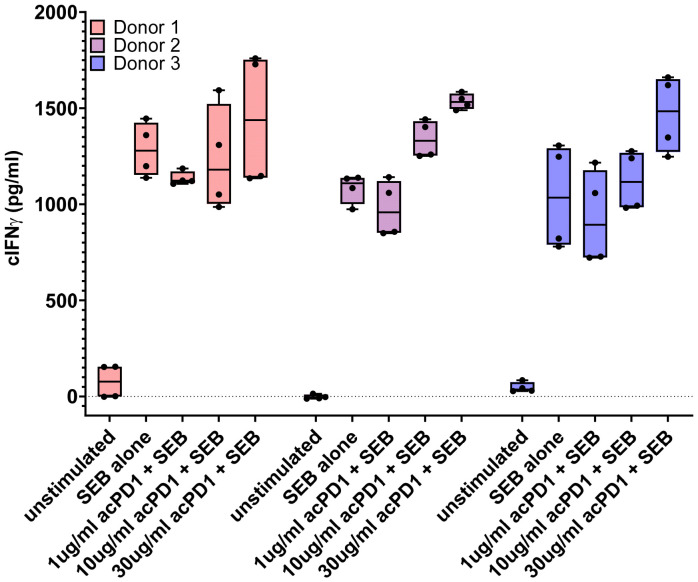
Effects of anti-canine PD-1 antibody on cIFN-γ production. Healthy canine PBMCs were stimulated with Staphylococcal enterotoxin B (SEB) and treated with increasing concentrations of anti-canine PD-1 antibody (acPD-1). cIFN-γ production is indicated in the y-axis. Individual ELISA reads shown.

In functional assays, stimulation of healthy cPBMCs with SEB induced robust IFN-γ production, which was further modulated by the addition of H9. Treatment with H9 consistently increased IFN-γ secretion in a dose-dependent manner. At higher concentrations (30 µg/mL), H9 enhanced SEB-driven IFN-γ responses, consistent with functional blockade of PD-1 signaling and its potential to modulate T-cell activity (Figure 2).

Next, the safety and tolerability of H9 was evaluated in a prospective, phase I non-randomized, unblinded, single-center trial in six companion dogs diagnosed with mammary tumors [32]. In that study, we demonstrated that IT administration of H9 was safe, tolerable, and had efficacy as a monotherapy. Remarkably, when IT H9 was combined with IT cowpea mosaic virus nanoparticle therapy, reduction in lung metastases was observed in two of the six companion dogs [32].

### 3.2. Generation and Characterization of Caninized Anti-Canine PD-1 Antibodies

Given the clinical impact of H9 against primary tumors and lung metastases in canine patients [32], we sought to optimize the therapeutic potential of the mouse anti-canine PD-1 H9 antibody by engineering a caninized version, designed to retain antigen specificity while minimizing immunogenicity.

**Figure 3:**
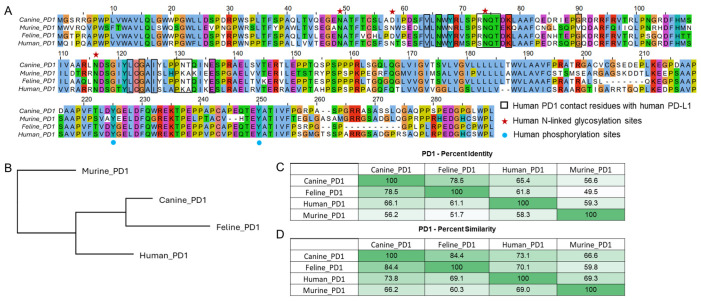
Cross-species PD-1 comparison. Sequence alignment of PD-1 from canine, feline, murine, and human, with amino acids colored using the Clustal coloring scheme to highlight conserved regions with similar chemical properties and variable regions where sequences differ across species (**A**). Human PD-1/PD-L1 contact residues (boxed), N-linked glycosylation sites (red stars), and phosphorylation sites (blue circles) are indicated. Phylogenetic tree illustrating the evolutionary relationship of PD-1 across these species (**B**). Cross-species percent identity representing the exact matches between sequences (**C**). Cross-species percent similarity accounting for substitutions for amino acids with similar biochemical properties (**D**).

Sequence alignments of canine, feline, human, and mouse PD-1 were used to assess sequence conservation and identify differences in functional regions across species [45] (Figure 3A). Notable amino acid differences in the functional region of PD-1 across species (canine PD-1 numbering) are V64, Y68, R73, G90, N131, and T132. Post-translational modifications characterized in human PD-1 are likely conserved at key N-linked glycosylation sites N49, N74, and N116, which are present across species and are involved in PD-L1 binding [46,47]. The N58 glycosylation site is only found in human and murine PD-1, where it also plays a role in PD-L1 binding. While phosphorylation sites at Y223 and Y248 (corresponding to Y225 and Y250 in canine PD-1) are involved in TCR signaling regulation, the extent and conservation of phosphorylation at these sites across species remain likely but not fully established [48]. A phylogenetic tree together with pairwise sequence identity and similarity of PD-1 proteins across species showed that canine and feline PD-1 are closely related, while human and murine PD-1 are more distantly related (Figure 3B–D). Together, these relationships provide insight into the structural and functional conservation of PD-1 across species, which may inform translational research and therapeutic applications.

**Figure 4:**
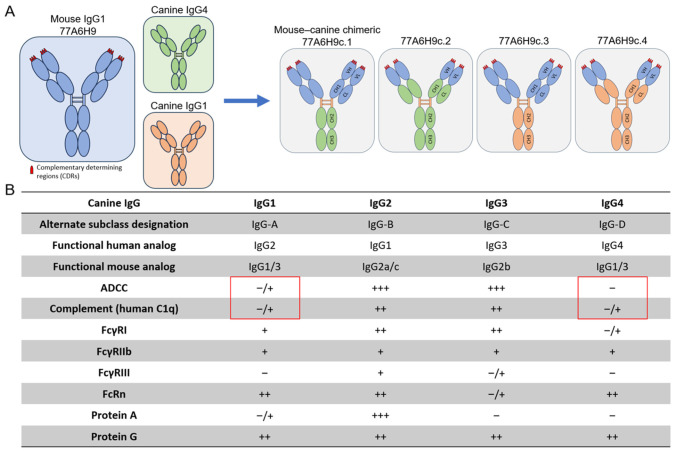
Caninization of mouse anti-PD-1 antibody. Schematic of the engineering of mouse–canine chimeric 77A6H9c antibodies (**A**). Activity profiles of canine IgG antibodies (**B**) (this information was adapted from Bergeron et al. and IMGT [49,50]). Red boxes highlight the functional activity of canine IgG1 and IgG4 subclasses. Binding and activation levels are represented as follows: “+++” for very high, “++” for good, “+” for moderate, “−/+” for minimal or negligible, and “−“ for no binding/activity. CL, constant light chain; CH, constant heavy chain; ADCC, antibody-dependent cellular cytotoxicity; FcγRI, Fc gamma receptor I; FcγRIIb, Fc gamma receptor IIb; FcγRIII, Fc gamma receptor III; FcRn, Fc neonatal receptor.

From the parent mouse antibody H9, four novel mouse–canine chimeric mAbs were engineered, 77A6H9c.1, 77A6H9c.2, 77A6H9c.3, and 77A6H9c.4, hereafter referred to as c.1, c.2, c.3, and c.4, respectively (Figure 4A). These chimeric antibodies retained the variable domains of the original mouse antibody while incorporating canine constant regions to improve compatibility with canine immune systems. The variable domains and, in some constructs, the constant heavy chain 1 (CH1) region of mouse antibody clone H9, were fused to either the hinge region (c.1 and c.3) or the CH1 domain of a canine IgG1 antibody (c.2 and c.4), which was further fused to a canine IgG4 Fc (c.1 and c.2) or IgG1 Fc (c.3 and c.4). It should be noted that both canine IgG1 and IgG4 elicit minimal Fc effector function (Figure 4B) [49,50], which is crucial to prevent the engineered antibodies from triggering immune cell targeting, and to function as immune checkpoint inhibitors rather than cytotoxic cPD-1-targeting antibodies [26]. This approach ensures that the antibodies can block immune checkpoints without promoting the clearance of PD-1-expressing cells through unwanted activation of effector immune cells.

Additionally, the canine IgG1 hinge was used in all four constructs over the canine IgG4 hinge to improve stability. Human IgG4 is known to participate in half-molecule exchange (Fab-arm exchange), resulting in asymmetrical, bispecific antibodies, where each half of the antibody originates from distinct parent antibodies [51,52]. The human S228 residue increases flexibility and allows rearrangement of disulfide bonds between cysteines. The canine IgG4 hinge has sequence similarities to the human IgG4 hinge region containing cysteines capable of forming inter-heavy-chain disulfide bonds (CPSC motif in human and CISPC in canine; Figure S3). Although the canine hinge includes an additional amino acid between these cysteines, which likely alters hinge geometry and reduces the potential for disulfide reshuffling and Fab-arm exchange, without experimentally validating its stability, we chose to avoid using the canine IgG4 hinge.

**Figure 5:**
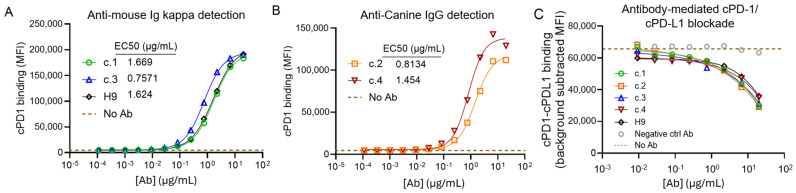
Anti-cPD-1 antibodies retain functional activity after caninization. Binding of the four caninized antibodies (c.1–c.4) and the parental H9 mouse antibody to cPD-1 with either anti-mouse Ig kappa detection for c.1 and c.3 antibodies (**A**) or anti-canine IgG (H + L) detection for c.2 and c.4 antibodies (**B**). Competition assay of caninized antibodies (c.1–c.4) and murine H9 blocking the interaction between cPD-1 and cPD-L1 (**C**). MFI, median fluorescence intensity; EC50, half maximal effective concentration; Ctrl, control; Ab, antibody.

The binding and competition assays of the engineered caninized anti-cPD-1 antibodies indicated that all four engineered antibodies (c.1–c.4) retained binding to cPD-1 with comparable levels to the original H9 mouse antibody (Figure 5A,B), demonstrating that the incorporation of canine constant regions did not disrupt retained antigen recognition. A competition assay using cPD-1-conjugated beads and soluble cPD-L1 confirmed that all four antibodies retained their ability to block the interaction between cPD-1 and cPD-L1 (Figure 5C). With increasing antibody concentration, the binding of cPD-L1 to cPD-1 decreased substantially, indicating effective competition. These results indicated that the caninized antibodies effectively block the cPD-1/cPD-L1 interaction in vitro, supporting their potential as immune checkpoint inhibitors. Based on expression levels (c.1 = 42.2 mg/L, c.2 = 2.0 mg/L, c.3 = 69.2 mg/L, c.4 = 10.3 mg/L) in CHO cells and canine IgG subclass functional activity profiles [49], antibody c.1 (hereafter referred to as HugPetmab), was chosen for further characterization and in vivo study.

### 3.3. Characterization of HugPetmab for In Vivo Studies

For in vivo studies, HugPetmab was produced as previously described and characterized as follows: An SDS-PAGE gel under nonreducing conditions showed the presence of a protein of approximately 180 kDa, slightly higher than the predicted molecular weight of 146.8 kDa. Multiple lower molecular weight bands were consistently observed before and after SEC-HPLC purification. These discrepancies in expected electrophoretic mobility could be attributed to altered mobility under SDS-denaturing, non-reducing conditions (Figure S4A). Similar banding patterns have been reported for IgG molecules and are commonly attributed to disulfide bond scrambling arising from thiol-disulfide exchange under non-reducing conditions [53]. LC-MS analysis of these bands indicates that the bands at ~180 kDa (Band 1) and ~75 kDa (Band 3) contain only the HC and LC at approximately a 1:1 ratio, consistent with 2HC:2LC and 1HC:1LC, respectively. The band at ~120 kDa (Band 2) contained only the HC, consistent with 2HC. The band at ~45 kDa (Band 4) contained only the LC, consistent with 2LC. The bands at ~120 kDa (Band 2) and ~45 kDa (Band 4) contained a small amount of the other antibody species (i.e., LC in the ~120 kDa band and HC in the ~45 kDa band) but at ~1% or less abundance relative to the main species (Table S1). Under reducing conditions, the antibody resolved into bands at approximately 50 kDa and 25 kDa, consistent with the expected heavy and light chains. DLS revealed a single clean peak corresponding to a hydrodynamic radius of 5.8 nm, consistent with the expected size of the antibody monomer (Figure S4B). The SEC-HPLC chromatogram revealed a single sharp peak with no other peaks visible, indicating that the antibody exists predominantly as a monomeric species, with no detectable aggregates or fragments (Figure S4C). LC-MS confirmed the expected antibody sequence of HugPetmab, confirming that the caninized construct retained the correct sequence and structural integrity after expression. Collectively, these results confirmed that the caninized antibody HugPetmab was structurally intact, monodisperse, and predominantly monomeric.

### 3.4. HugPetmab Is Safe and Controls Tumor Growth

The primary objective of the in vivo study was to confirm the safety of administration of HugPetmab with secondary observations on tumor responses during the four weeks of weekly IT therapy. Most of the dogs treated had a single tumor, except for P3, a CMC patient with two treated tumors, and P7, an MCT patient with three treated tumors (Table 1). The hemogram studies of treated patients showed minor fluctuations in erythrocytes, hemoglobin, and hematocrit in two patients (P1 and P7) as well as minor fluctuations in leukocytes in a few dogs (P1, P2, and P7). These fluctuations were transient and related mostly to the disease, but not to acPD-1 therapy (Table S2). Furthermore, the QOL reported good to excellent status of the treated patients (Table S2). These findings suggest that HugPetmab therapy at the doses used is safe and well-tolerated, and corroborated prior safety studies using the parent mouse anti-cPD-1 antibody H9 [32].

**Figure 6:**
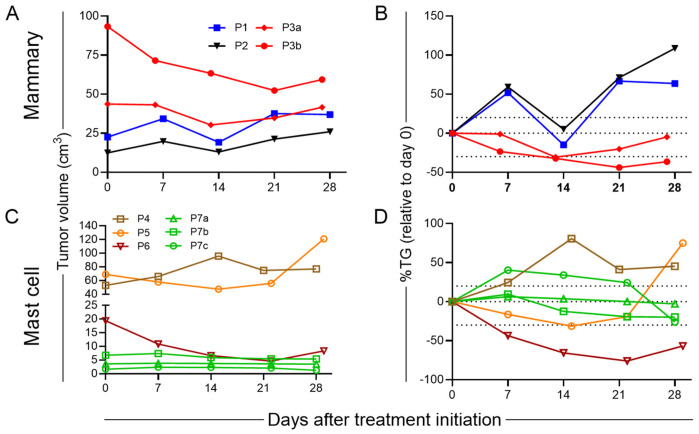
Intratumoral HugPetmab treatment in canine cancer patients. Absolute (**A**,**C**) and relative (**B**,**D**) tumor volumes over time are shown for canine mammary tumor patients P1–P3 (**A**,**B**) and mast cell tumor patients P4–P7 (**C**,**D**). Treatments were at D0, D7, D14 and D21, with some patients receiving doses +/− 1–2 days from the scheduled treatment dates. The percentage of change in tumor growth (%TG; relative to D0; **B**,**D**) indicates stable disease in the dotted areas (−30% to 20%), above 20% indicates progressive disease and below −30% indicates partial response.

The treated tumors in CMC patients P1 and P2 showed fluctuating tumor sizes, with periods of growth and reduction in tumor size before ultimately progressing, and in patient P3, regression was observed in one tumor and tumor control in the other (Figure 6A). These responses represent PD in patients P1 and P2, and PR and SD in patient P3′s tumors (Figure 6B).

The IT HugPetmab therapy in MCT patients showed tumor progression in patient P4, tumor control followed by tumor growth in patient P5, tumor reduction in patient P6, and tumor control in the three tumors in patient P7 (Figure 6C). These responses represent PD in P4, SD and PD in P5, PR in P6, and SD in P7 (Figure 6D). Regression analysis did not show significant changes in tumor volume (Table S3), with only a trend towards significance in T1 of patient P3 and T2 of patient P7 (*p* = 0.055, *p* = 0.067; Table S3).

The clinical benefit, defined as SD + PR + CR, was 43% (3/7 dogs; 1/3 in CMC, and 2/4 in MCT). However, if counted by the number of tumors treated, it was 60% (6/10 tumors; two CMC and four MCT; Figure 6B,D). The findings from this pilot trial suggest that HugPetmab is safe and well-tolerated and provide preliminary insights into its therapeutic activity.

## 4. Discussion

PD-1 blockade has dramatically changed the treatment paradigm across human solid tumors [54,55,56,57,58]. Given the remarkable responses, immunotherapy in veterinary medicine is slowly developing with increasing numbers of published immunotherapeutic agents [19,20,21,22,23,24,25,26,27,28,29,30,31,59,60,61,62], and a few clinical studies reported with anti-PD-1 [22,63,64]. Mizuno’s group reported safety and efficacy of systemically administered rat–canine chimeric and caninized anti-PD-1 antibodies in 30 dogs with oral malignant melanoma (OMM) as well as in other spontaneous tumors, including two CMC cases [22]; a case report describing successful systemic anti-PD-1 treatment in two canine oral adenocarcinoma patients [63], and in 37 non-OMM patients [64]. A case report described successful systemic anti-PD-1 treatment in a canine oral adenocarcinoma patient [65], and a pharmacokinetic study in four healthy dogs suggested that treatment with a caninized anti-PD-1 antibody was safe and well-tolerated [26]. Although most of the canine cancer patients previously received various treatments, clinical responses were observed along with irAEs as observed in humans [66].

It should be noted that in previous clinical studies canine cancer patients receive systemic (intravenous; IV) anti-PD-1 therapy, which likely contributed to the irAEs observed in treated patients [22,63,64,65]. Systemic anti-PD-1 therapy has been associated with irAEs, including therapy-related death in both human and canine cancer patients [22,66,67]. In contrast, IT treatment has not been associated with serious irAEs in either human cancer patients [68,69] or in canine patients treated with anti-PD-1 [32], IL2/IL12 [59,60,62], or anti-OX40 combined with a toll-like receptor (TLR3/8) agonist [61]. While we cannot rule out systemic toxicity if HugPetmab therapy would be applied via IV, IT therapy requires low doses of immunotherapeutic agents, which leads to low or no biological toxicities and alleviates financial and logistical challenges associated with the large doses needed to systemically treat dogs [68,69].

In this study we reported on production, in vitro reagent characterization, and in vivo treatment responses of a mouse anti-canine PD-1 monoclonal (H9) antibody, which was previously evaluated in CMC patients with good safety, tolerability and efficacy as a monotherapy or when combined with a plant viral nanoparticle [32]. The chimeric mouse–canine anti-canine PD-1 (HugPetmab) antibody developed on the H9 backbone and characterized in this study blocked the interaction between cPD-1 and cPD-L1 in vitro, suggesting its potential as an immune checkpoint inhibitor. Furthermore, HugPetmab as a monotherapy in canine patients indicated safety, tolerability, and tumor growth control in most of the injected tumors, supporting the potential therapeutic activity of HugPetmab. Development of this antibody will enable research of anti-PD-1 therapy to promote anti-tumor immunity in canine cancer patients.

We did not measure the presence of antibody drug antibodies (ADAs) because (i) there is not a validated assay; (ii) in the previous study we saw efficacy in two dogs treated up to 16 weeks without any irAEs [32]; and (iii) in this study, as well as in other canine studies [22,63,64] and human studies [70], the presence of ADAs does not substantially affect treatment with anti-PD-1/PD-L1 immunotherapy. The antibody was caninized to reduce potential immunogenicity and enable long-term dosing in future studies. We recognize the importance of ADA assessment, and we plan to incorporate validated ADA assays in future studies with a larger patient cohort.

While these findings are encouraging, several limitations should be noted. The current study included a small number of patients. Due to the small number of patients in this study, it was not feasible to include a randomized control group, and the follow-up period was relatively short. In addition, pharmacokinetic and pharmacodynamic analyses were not performed, limiting mechanistic insights of antibody performance in vivo. These limitations underscore the need for larger controlled studies to better define the safety, efficacy, and mechanistic effects of HugPetmab in canine cancer patients.

Future studies will also aim to evaluate the efficacy of anti-cPD-1 in combination with chemotherapy or other available immune checkpoint inhibitors including aCTLA-4 [29,30,31], aPD-L1 [20,21,23,24,25,28], IL2/IL12 [59,60,62] and OX40 [61] to improve outcomes in canine cancer patients. As we demonstrated changes in the tumor microenvironment induced by H9 and combined with the viral nanoparticles using NanoString arrays [32], we envision that HugPetmab will serve as an important tool to investigate correlative biomarkers of response and mechanisms of resistance to PD-1 checkpoint therapy in immune competent pet dogs. Given the striking biologic, immunologic, and clinicopathologic similarities with human tumors, future clinical studies in canine cancer patients with HugPetmab alone or combined with available immunotherapies could inform human clinical trial design.

## 5. Patents

CJH, MEA, RJN, PS, JLR, and HAP have submitted patent applications related to the subject matter in this publication.

## Figures and Tables

**Table 1:** Clinicopathologic characteristics of enrolled patients.

Patient	Age, y.	Weight, kg	Size; cm	Histopathologic Type	Clinical Stage	Histo Grade	Breed	Spayed/Neutered
CMC patients								
P1	11	10.0	4.7 × 3.1	Tubular carcinoma	IV	III	Beagle	F/No
P2	8	9.2	4.2 × 2.5	Complex carcinoma	I	I	Boston Terrier	F/Yes
P3a	14	2.8	4.7 × 4.3	Invasive papilar carcinoma	IV	II	Chihuahua	F/No
P3b			10.9 × 4.1	Complex carcinoma	IV	II		
MCT patients								
P4	10	10.0	4.8 × 4.7	Low grade */Grade 2 †	III	II	Pug	M/Yes
P5	6	35.0	6.0 × 4.8	Low grade */Grade 1 †	III	II	Mongrel	F/Yes
P6	4	23.4	4.2 × 3.1	Low grade */Grade 1 †	I	II	Boxer	F/No
P7a	11	8.5	2.5 × 1.7	Low grade */Grade 1 †	III	II	Schnauzer	F/Yes
P7b			2.8 × 2.2	Low grade */Grade 1 †				
P7c			2.4 × 1.2	Low grade */Grade 2 †				

Abbreviations: y, years; kg, kilograms; cm, centimeters; Histo, histopathological tumor grade; CMC, canine mammary tumors; MCT, mast cell tumor. F, female; M, male. Stage I: T1 N0 M0; Stage II: T2 N0 M0; Stage III: T2 N1 M0 or T3 N0 M0; Stage IV: Tn Nn M1. T1: tumor ≤ 2 cm in diameter; T2: tumor 2–4 cm in diameter, T3: tumor > 4 cm in diameter; N0: no evidence of regional lymph node involvement, N1: histologic/cytologic evidence of regional lymph involvement, N2: fixed nodes; M0: no evidence of distant metastasis, M1: evidence of distant metastasis. *, †, histopathological classification according to Kiupel and Patnaik classifications, respectively.

## Data Availability

The data and materials are available from the corresponding author upon reasonable request.

## Supplementary Materials

The following supporting information can be downloaded at: https://www.mdpi.com/article/10.3390/cells15110966/s1, Figure S1: Isolation of H9 antibody. Monoclonal antibodies were generated via conventional hybridoma procedures using Balb/C mice immunized with the extracellular domain (ECD) of recombinant canine PD-1 (cPD-1). Splenocytes were isolated from the immunized mice and then fused with SP2/0 myeloma cells. Cell fusions were performed by electrofusion and plated onto 96 well plates. Supernatants were screened by ELISA with PD-1 ECD protein and by flow cytometry using CHO cells that overexpressed canine PD-1. From this screening, five individual PD-1 monoclonal antibodies were isotyped and prepared as purified IgG for binding and functional studies. Clone 77A6H9 was selected as the lead antibody; Figure S2: Characterization of H9 antibody. Representative SDS-PAGE image showing the presence of H9 at 150 kDa (A); dynamic light scattering indicates the presence of a single peak around 5.4 nm with an estimated molecular weight by radius (Mw-R) of 175.3 kDa (B), and SEC-HPLC shows a single peak with a 99.6% purity (inset on the top left; and the single peak without standards on the top right inset) (C). The y-axis refers to milli absorbance units (mAU) of H9 by the detector and the x-axis refers to the retention time for H9. Protein standards were used to estimate molecular weight (MW, in kilo Daltons; kDa), conformation, and retention time (RT) of H9. R, reducing; NR, non-reducing; Figure S3: Alignment of human and canine hinge region of IgG antibodies. Cysteines highlighted in red boxes indicate residues that form interchain disulfide bonds in human IgG4 and potentially in canine IgG4, contributing to Fab-arm exchange instability; Figure S4: Characterization of caninized antibody HugPetmab. Representative SDS-PAGE gel of HugPetmab. LC-MS revealed Bands 1 (~180 kDa) and 3 (~75 kDa) contain both the heavy and light chains at an approximate ratio of 1:1. Band 2 (~120 kDa) contains the heavy chain; Band 4 (~50 kDa) contains the light chain. DLS imaging indicates the presence of a single peak around 5.8 nm with an estimated MW of 206.8 kDa (B), and SEC-HPLC chromogram shows a single peak with molecular weight of 182.7 kDa with 94.6% purity (inset on the top left; and the single peak without standards is on the top right inset) (C). Protein standards were used to estimate molecular weight (MW), conformation, and retention time (RT) of HugPetmab. R, reducing, NR, non-reducing; Mw-R, molecular weight by radius; mAU refers to milli absorbance units or absorption of HugPetmab by the detector; and the X-axis (time, min) refers to the retention time for HugPetmab; Table S1: MS_SDS PAGE bands; Table S2: Hemograms and quality of life of treated canine cancer patients; Table S3: Tumor volume changes in treated patients.

## Abbreviations

%TG: percentage of change in tumor growth; ADAs: antibody drug antibodies; AmBic: ammonium bicarbonate; CH1: constant heavy chain 1; CHO cells: Chinese hamster ovary cells; cIFN-γ: canine interferon gamma production; CMC: canine mammary cancer; CMT: canine mammary tumors; Con A: concanavalin A; CR: complete response; CTLA-4: cytotoxic T-lymphocyte- antigen-4; cPBMCs: canine peripheral blood mononuclear cells; cPD-1: canine programmed cell death 1; DLS: dynamic light scattering; ECD: extracellular domain; ELISA: enzyme-linked immunoassay; HC: heavy chain; HEK293: human embryonic kidney; HPLC: high-performance liquid chromatography; ICI: immune checkpoint inhibitor; IFN-γ: Interferon gamma; IHC: immunohistochemistry; IT: Intratumoral; irAEs: immune-related adverse events; LC: light chain; LC-MS: liquid chromatography tandem mass spectrometry; OMM: oral malignant melanoma; PBMC: peripheral blood mononuclear cell; PBS: phosphate-buffered saline; PD: progressive disease; PD-1: programmed cell death 1; PD-L1: programmed death-ligand 1; PR: partial response; QOL: quality of life; RACE: rapid amplification of cDNA ends; SD: stable disease; SEB: staphylococcal enterotoxin B; SEC: size-exclusion chromatography; SDS-PAGE: Sodium Dodecyl Sulfate-Polyacrylamide Gel Electrophoresis; SP3: single-pot solid-phase-enhanced sample preparation; TLR: toll-like receptor; TME: tumor microenvironment; Tv: tumor volume.
