# Supplementary material for: Characterization of Anti-Canine PD-1 Antibodies
Source: Cells. 2026 May 23;15(11):966. doi: 10.3390/cells15110966 (PMC13256597; doi:10.3390/cells15110966)
Supplement: Supplementary file 1 [file cells-15-00966-s001.zip › Suppl figures revision.pptx]

## Slide 1
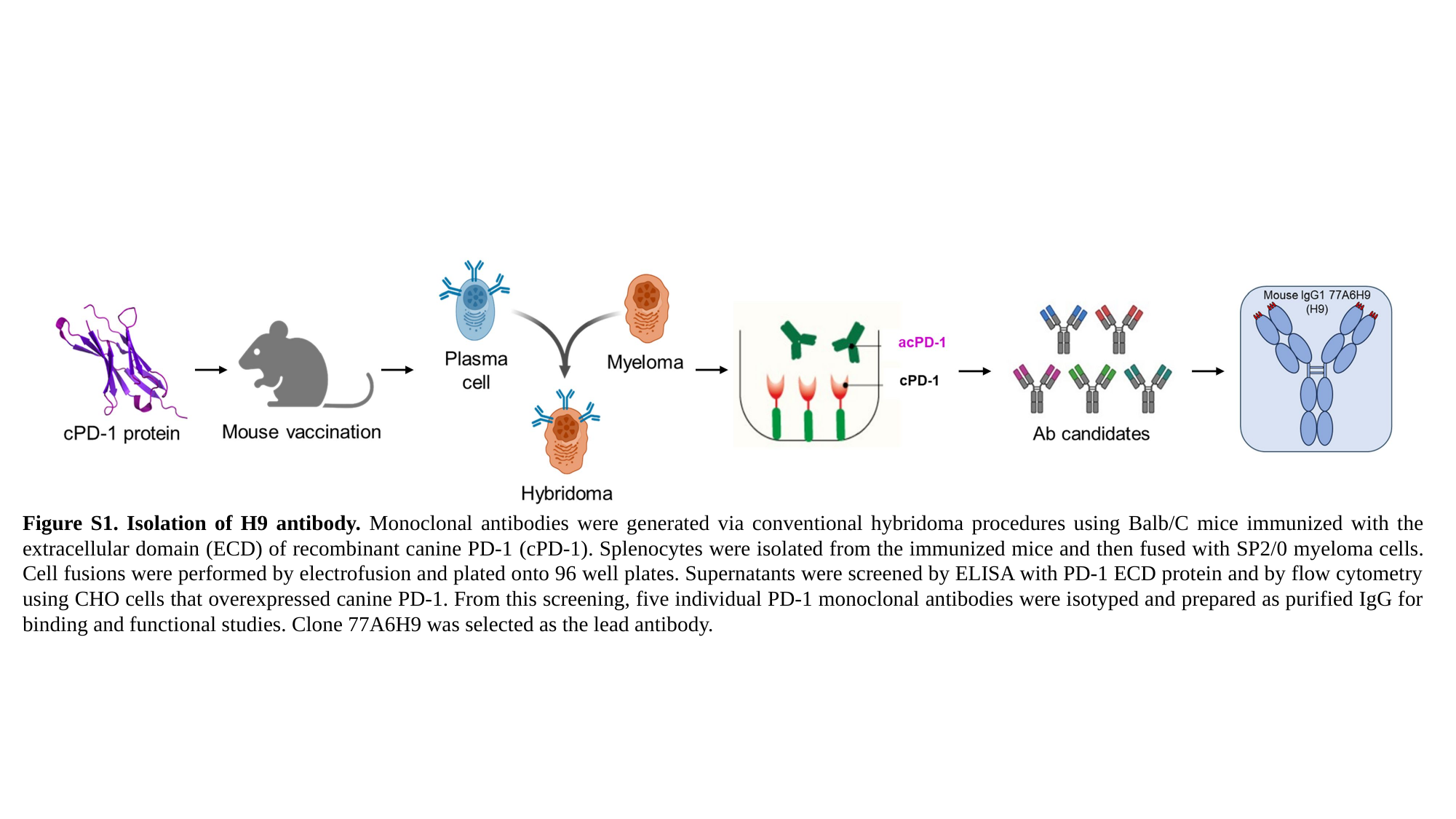

Figure S1. Isolation of H9 antibody. Monoclonal antibodies were generated via conventional hybridoma procedures using Balb/C mice immunized with the extracellular domain (ECD) of recombinant canine PD-1 (cPD-1). Splenocytes were isolated from the immunized mice and then fused with SP2/0 myeloma cells. Cell fusions were performed by electrofusion and plated onto 96 well plates. Supernatants were screened by ELISA with PD-1 ECD protein and by flow cytometry using CHO cells that overexpressed canine PD-1. From this screening, five individual PD-1 monoclonal antibodies were isotyped and prepared as purified IgG for binding and functional studies. Clone 77A6H9 was selected as the lead antibody.

## Slide 2
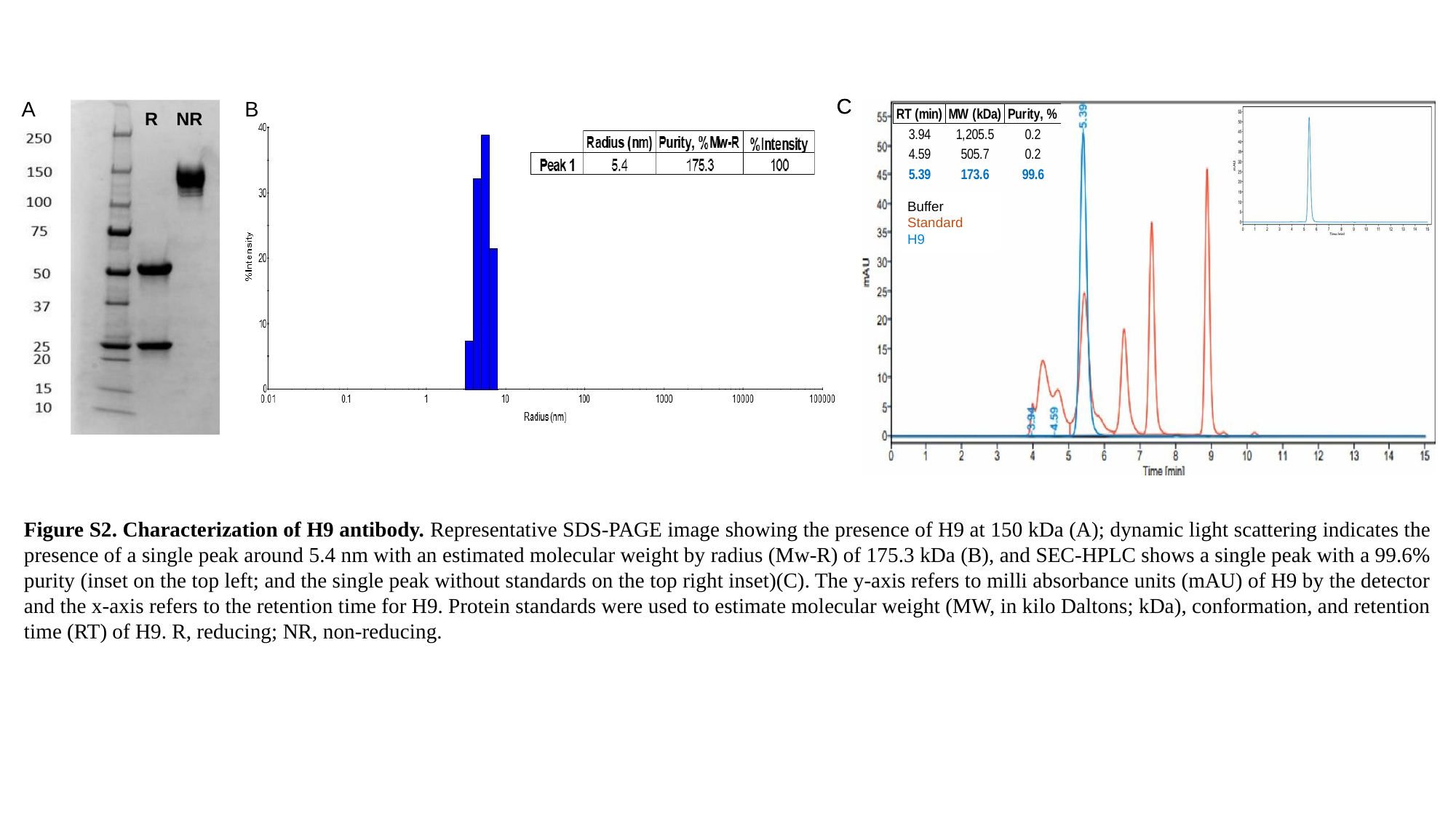

C
C
Buffer Standard H9
B
A
R
NR
Figure S2. Characterization of H9 antibody. Representative SDS-PAGE image showing the presence of H9 at 150 kDa (A); dynamic light scattering indicates the presence of a single peak around 5.4 nm with an estimated molecular weight by radius (Mw-R) of 175.3 kDa (B), and SEC-HPLC shows a single peak with a 99.6% purity (inset on the top left; and the single peak without standards on the top right inset)(C). The y-axis refers to milli absorbance units (mAU) of H9 by the detector and the x-axis refers to the retention time for H9. Protein standards were used to estimate molecular weight (MW, in kilo Daltons; kDa), conformation, and retention time (RT) of H9. R, reducing; NR, non-reducing.

## Slide 3
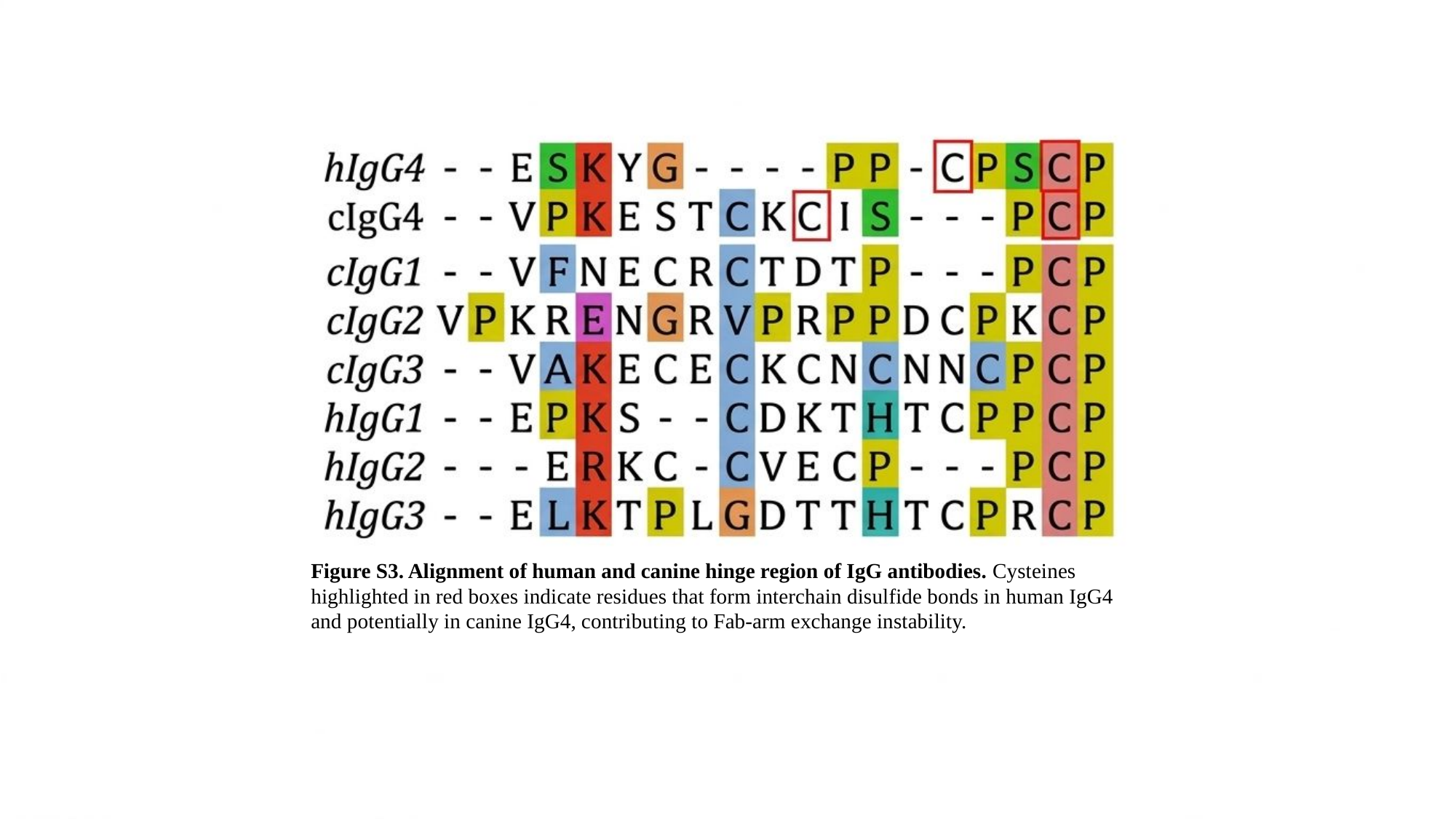

Figure S3. Alignment of human and canine hinge region of IgG antibodies. Cysteines highlighted in red boxes indicate residues that form interchain disulfide bonds in human IgG4 and potentially in canine IgG4, contributing to Fab-arm exchange instability.

## Slide 4
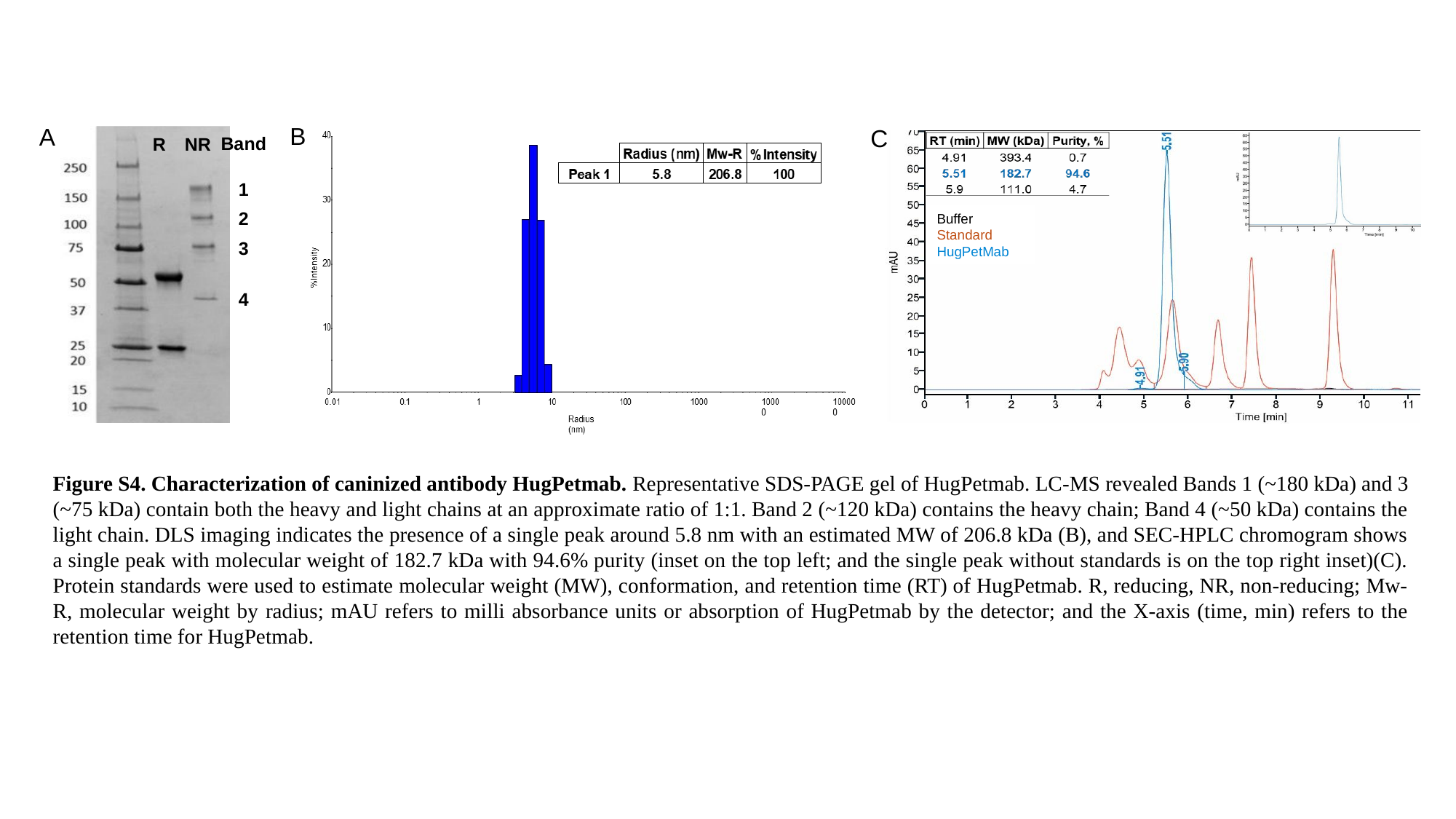

B
A
R
NR
Band
1
2
3
4
C
Buffer Standard HugPetMab
Figure S4. Characterization of caninized antibody HugPetmab. Representative SDS-PAGE gel of HugPetmab. LC-MS revealed Bands 1 (~180 kDa) and 3 (~75 kDa) contain both the heavy and light chains at an approximate ratio of 1:1. Band 2 (~120 kDa) contains the heavy chain; Band 4 (~50 kDa) contains the light chain. DLS imaging indicates the presence of a single peak around 5.8 nm with an estimated MW of 206.8 kDa (B), and SEC-HPLC chromogram shows a single peak with molecular weight of 182.7 kDa with 94.6% purity (inset on the top left; and the single peak without standards is on the top right inset)(C). Protein standards were used to estimate molecular weight (MW), conformation, and retention time (RT) of HugPetmab. R, reducing, NR, non-reducing; Mw-R, molecular weight by radius; mAU refers to milli absorbance units or absorption of HugPetmab by the detector; and the X-axis (time, min) refers to the retention time for HugPetmab.
