# Supplementary material for: Characterization of Anti-Canine PD-1 Antibodies
Source: Cells. 2026 May 23;15(11):966. doi: 10.3390/cells15110966 (PMC13256597; doi:10.3390/cells15110966)
Supplement: Supplementary file 1 [file cells-15-00966-s001.zip › Supplemental File.pdf]

Supplemental file

Canine patient recruitment and inclusion criteria

Male or female companion patients with at least one solid tumor mass with the longest measurement greater than two cm were recruited. This size cut-off is related to a direct correlation between the malignant nature of mammary tumors and the size; a mass smaller than this defined size could result in enrolling patients with benign tumors [1,2]. Inclusion criteria at diagnosis included absence of metastatic dissemination based on thoracic radiography and abdominal ultrasound; no severe infection or ulceration of the mammary target tumor; no chronic life-threatening disease or any systemic disease that could influence the immune system response (such as endocrinopathies, immune-mediated disease, leishmaniasis and ehrlichiosis); no treatment with immunosuppressive drugs; absence of clinical signs compatible with inflammatory mammary carcinoma (as pain, warmth, erythema); and prior chemotherapy, radiation therapy, or other investigational drugs were not allowed. For this trial, we followed the ARRIVE guidelines 2.0 [3] (supplemental file 1), and, whenever possible, we followed the guidelines from the "Human Intratumoral Immunotherapy Expert Recommendations" [4].

Surgical procedures

All incisional biopsies were collected under sedation (medetomidine 10 μg/kg and methadone 0.3 mg/kg, both intramuscularly). Analgesia was provided after the procedure with tramadol (3 mg/kg orally every 12 h for four days) and gabapentin (10 mg/kg orally every 12 h for four days). If the histopathological evaluation of the incisional biopsy evidenced a non-malignant epithelial mammary tumor, the patient was removed from the study. Intratumoral injections were performed under topical local anesthesia using a tetracaine ointment.

Four weeks after the first HugPetmab treatment, canine patients were surgically treated as follows: regional mastectomy was performed when the largest diameter of the injected tumor was smaller than three or five cm in dogs with a weight lower or greater than 10 kg, respectively, and complete unilateral mastectomy when it was higher than three or five cm (if dog's weight was lower or greater than 10 kg, respectively) or if multiple nodules were present along the mammary chain. For cutaneous mastocytomas surgeries, all tumors were removed with 1.5 cm lateral surgical margins and a deep plane.

For surgical procedures, all patients received medetomidine and methadone (10 and 300 micrograms/kg, intramuscularly, respectively), followed by induction with propofol (1 mg/kg, intravenously) and inhalational anesthesia with isoflurane (1.5-2.5%). Intravenous cephazolin was given 20 min before surgery (22 mg/kg). Further, depending on the mastectomy procedure, transversus abdominis plane block with bupivacaine (up to 2 mg/kg) and/or epidural anesthesia using morphine (0.1 mg/kg) plus bupivacaine (up to 2 mg/kg) was provided. Diffusion catheters were placed during surgery (DC Mila International Inc) in order to administer bupivacaine (1-2 mg/kg every 6 h) in the post-operative period. Catheters were left in place for 3 days. Soft sterile wound dressings and a tubular mesh were placed to cover the wound. Post-surgical therapy also included firocoxib (5 mg/kg, orally every 24 h for seven days) and tramadol (3 mg/kg, orally every 12 h for three days). No post-operative antibiotics were prescribed. The wounds healed uneventfully, and skin sutures were removed after 12 days.
